# Reply: Inaccurate measures of outcomes in the two-sample Mendelian randomization of vitamin D with miscarriage

**DOI:** 10.1093/hropen/hoae026

**Published:** 2024-04-27

**Authors:** Feng Zhang, Jingtao Huang, Gangting Zhang, Mengyang Dai, Tailang Yin, Chunyu Huang, Jue Liu, Yan Zhang

**Affiliations:** Reproductive Medical Center, Renmin Hospital of Wuhan University, Wuhan, China; Department of Clinical Laboratory, Institute of Translational Medicine, Renmin Hospital of Wuhan University, Wuhan, China; Reproductive Medical Center, Renmin Hospital of Wuhan University, Wuhan, China; Wuhan Meizhao Health Management Co, Ltd, Wuhan, China; Reproductive Medical Center, Renmin Hospital of Wuhan University, Wuhan, China; Reproductive Medical Center, Renmin Hospital of Wuhan University, Wuhan, China; Shenzhen Key Laboratory for Reproductive Immunology of Peri-implantation, Shenzhen Zhongshan Institute for Reproduction and Genetics, Shenzhen Zhongshan Obstetrics & Gynecology Hospital (formerly Shenzhen Zhongshan Urology Hospital), Shenzhen, China; Department of Epidemiology and Biostatistics, School of Public Health, Peking University, Beijing, China; Department of Clinical Laboratory, Institute of Translational Medicine, Renmin Hospital of Wuhan University, Wuhan, China

Sir,

We thank Yang and colleagues for their interest in our recent article (Zhang *et al.*, 2024). Having carefully reviewed their letter (Yang *et al.*, 2024), we found it very insightful, and we would like to address each of the questions raised in turn.

Concerning Yang *et al.*’s first comment that ‘Women having “zero” miscarriage were actually those who had at least one stillbirth or termination but no miscarriage’; those women currently categorized into ‘zero’ would be expected to have lower 25-hydroxyvitamin D (25OHD) levels than they should have, i.e. ‘true zeros’. Given the current lack of strong evidence suggesting that women who have experienced a stillbirth or termination have lower 25OHD levels, we believe it is important to exercise caution in making assumptions.

Regarding the comments raised by Yang *et al.* concerning the effect of 25OHD on number of miscarriages as ‘Beta’ without any clear units, considering that the number of miscarriages is a continuous variable, we have chosen beta as the outcome. The limited unit information provided in the GWAS summary data has resulted in beta values without a clear unit (Revez *et al.*, 2020). Recognizing this limitation, and in order to enhance the clinical interpretability of the results (similar to a previous study (Yuan *et al.*, 2023)), we have described the findings as follows in our original text (Zhang *et al.*, 2024): ‘The primary IVW analysis indicated that for each one-unit increase in genetically determined natural-log-transformed serum 25OHD concentration, there was little causal association with decreased odds of miscarriage (odds ratio (OR) = 0.995, 95% CI: 0.888 to 1.114, *P* = 0.927; Fig. 2), nor with the number of miscarriages (β = –0.004, 95% CI: –0.040 to 0.032, *P* = 0.829; Fig. 3)’.

Yang and colleagues also highlighted that ‘In FinnGen, GWAS of pregnancy and perinatal disorders, including miscarriage, are in all women, meaning that those who have never been pregnant, and therefore could not experience a miscarriage, are included as controls’ (Yang *et al.*, 2024). We think they have a valid point, and it could indeed cause some interference with the results of our study. Unfortunately, owing to our limited access to only GWAS summary data and not individual-level data, we were unable to exclude women who have never been pregnant from the control group.

Yang *et al.*’s concerns primarily center around the accuracy and reliability of the GWAS summary data, which we also acknowledge and have addressed in the discussion of our article (Zhang *et al.*, 2024). We had hoped to examine the original data sources; however, unfortunately, we were unable to access these resources. We look forward to a resolution of this issue in the future to advance research utilizing GWAS data, including Mendelian randomization studies.

Given the potential limitations of some of the GWAS data in our study, we have included both 25OHD concentration and vitamin D deficiency as exposures, as well as miscarriage and number of miscarriages as outcomes in our analysis simultaneously, with the aim to enhance the reliability of our results.

To conclude, as Mendelian randomization studies are flourishing, taking the time to reflect on and improve methodological limitations is very beneficial. We share Yang *et al.*’s concerns in this regard and hope that with methodological advancements and increased accessibility to original individual-level data, the above-mentioned issues can be more effectively addressed in the future. We also call for more global studies on the efficacy and safety of vitamin D supplementation and its relationship with miscarriage.
